# Estimation of soil salt content in the oasis tillage layer based on hyperspectral transformation and model combination

**DOI:** 10.1371/journal.pone.0347859

**Published:** 2026-04-30

**Authors:** Yanping Guo, Xuemei Wang, Dun Li, Kunyu Li, Qian Zhang

**Affiliations:** 1 College of Geographic Sciences and Tourism, Xinjiang Normal University, Urumqi, China; 2 Xinjiang Laboratory of Lake Environment and Resources in Arid Zone, Urumqi, China; University of Queensland - Saint Lucia Campus: The University of Queensland, AUSTRALIA

## Abstract

Soil salinization poses a serious threat to global soil health and agricultural productivity, especially in arid and semi-arid regions, making the accurate assessment of its extent and severity crucial. This study employs hyperspectral remote sensing data to estimate soil salinity content (SSC) in the Weigan-Kuqa River Oasis in Xinjiang, China. To address hyperspectral dimensionality and noise challenges, multiple spectral transformation methods are systematically introduced and compared, including mathematical transformations, continuous wavelet transformation (CWT), discrete wavelet transformation (DWT), and their combined approaches. By incorporating multiple machine learning algorithms—including random forest (RF), support vector machine (SVM), gradient boosting decision tree (GBDT), and deep forest (DF)—a novel integrated framework that combines multi-transformation with multi-model algorithms for estimating SSC was developed. Results revealed that *R*-DWT showed the strongest correlation with SSC (|*r*|_max_ = 0.621). SSC-sensitive bands are primarily distributed across the absorption regions of 1633 nm (clay minerals), 1809–1810 nm and 1951–1955 nm (hydrated ions), 1969–1971 nm and 1987–1989 nm (crystalline water and hydroxyl groups), and 2001–2041 nm (soluble salts). Among the spectral transformations, (1/*R*)′-CWT-2^7^ yielded relatively high prediction accuracy. At the modeling algorithm level, the DF algorithm exhibited superior overall performance compared with the other algorithms. Among all models, the *R*-DWT-H7-DF model achieved the best overall performance, with *R*² values of 0.87 for the training set and 0.67 for the test set. Research demonstrates that integrating appropriate spectral transformations with modeling methods can enhance the accuracy of SSC estimation, providing a feasible technical pathway and methodological support for monitoring soil salinization in arid regions.

## Introduction

High soil salt content (SSC) contributes to soil salinization, which impairs soil properties and crop productivity [1]. Particularly in arid and semi-arid zones, intense evaporation coupled with limited precipitation has intensified soil salinization, giving rise to a range of ecological and agricultural issues [2]. Timely acquisition and accurate assessment of the severity, spatial extent, and distribution of soil salinization are essential prerequisites for effective reclamation of saline soils and prevention of further land degradation [3]. Traditional SSC determination methods primarily rely on extensive manual field sampling and laboratory chemical analyses. However, this approach entails high monitoring costs, limited spatial coverage, destructive sampling, and poor representativeness [4]. In contrast, remote sensing technology, with its advantages of large-scale coverage and real-time monitoring, has demonstrated substantial value in mapping and dynamically monitoring salinized soils [5]. Optical remote sensing (such as Landsat and Sentinel series) offers high temporal and spatial resolution and can indirectly derive soil salinity information from spectra or vegetation indices [6,7]. Microwave remote sensing can achieve all-weather, day-and-night imaging and has a certain penetration ability, enabling the monitoring of salinity changes under conditions where optical sensing is limited [8]. Hyperspectral remote sensing acquires continuous and rich spectral information through narrow bands, and can capture subtle differences in soil spectral characteristics compared to multispectral and microwave remote sensing, thereby achieving rapid, non-destructive, and precise detection of soil physical and chemical parameters [9,10]. However, hyperspectral data contain numerous bands spanning a wide wavelength range, resulting in data redundancy and collinearity among adjacent bands. This increases the complexity of subsequent modeling and may lead to overfitting and decrease accuracy [11]. Therefore, effective extraction of key spectral information and construction of robust models remain current technical challenges [12,13]. In early studies, researchers employed traditional methods, such as continuum removal (CR), multivariate scatter correction (MSC), and standard normal variate (SNV), to eliminate background noise and scattering effects, thereby enhancing spectral features [14,15]. Subsequently, mathematical transformations including reciprocal (1/*R*), logarithmic (lg*R*), logarithm of reciprocal (lg(1/*R*)), and differential transformations were introduced into hyperspectral data processing. These operations aim to amplify inter-band differences, mitigate background interference, and counteract baseline drift, thereby improving the discrimination of characteristic spectral bands [16–18]. Xiong et al. [19] and Chang et al. [20] evidenced that differential transformation accentuates spectral peaks more effectively than original or non-differential spectra, thereby improving the identification of subtle differences and facilitating more efficient feature extraction. Hou et al. [21] reported that the first-order differentiation (FD) surpasses the second-order differentiation (SOD) and other transformations in enhancing soil reflectance disparities, mitigating spectral noise and particle size effects, and boosting model predictive capabilities. As a commonly used technique in hyperspectral data processing, integer-order differentiation has shown excellent performance in spectral feature enhancement and soil property inversion. However, the step size of integer-order differentiation is relatively large, which may cause the loss of some useful information during processing [22]. In recent years, fractional-order differentiation (FOD) has been widely studied as an extension of integer-order differentiation in hyperspectral data processing and surface parameter inversion. By introducing a continuously adjustable order parameter, FOD can refine spectral reflection information within a smaller range of order changes, thereby enhancing spectral detail features and suppressing background noise [23]. However, existing studies have shown that the performance of FOD is highly sensitive to the order selection, and not all orders can achieve ideal results. High-order FODs may be more sensitive to noise in practical applications, thereby affecting the stability and prediction accuracy of the model [24]. Some studies have also indicated that in certain cases, the FD effect in integer-order differentiation is even better than FOD, and can significantly improve the correlation between the spectrum and SSC [25]. This suggests that in specific applications, integer-order differentiation still has reliability and stability, and can provide an effective foundation for feature extraction. With the advancement of spectral technologies, wavelet transformation (WT) has demonstrated notable advantages in hyperspectral data processing [26]. In contrast to conventional transformations, continuous wavelet transformation (CWT) and discrete wavelet transformation (DWT) excel in spectral smoothing, denoising, and extracting weak signals, leading to substantial improvements in inversion model accuracy [27,28]. Recent studies have attempted to integrate mathematical transformations and wavelet transformations for spectral processing. Results showed that combining FD with either CWT or DWT significantly strengthens the correlation between spectra and soil organic carbon (SOC) content, yielding higher prediction accuracy than using either FD or WT alone [29,30]. These findings indicate that both FD and WT can enhance spectral feature expression and improve model performance. Nevertheless, the combined application of FD and WT in estimating SSC remains underexplored. Thus, further in-depth research is required to identify the optimal spectral transformation method.

In recent decades, machine learning (ML) and deep learning (DL) algorithms have exhibited remarkable superiority in a wide range of modeling tasks, leveraging their powerful nonlinear modeling capability and robustness to noise [31]. Among ML techniques, the support vector machine (SVM) stands out for its exceptional generalization ability, rendering it especially adept at handling high-dimensional datasets with limited samples. Nevertheless, SVM performance can be compromised by noise sensitivity and the curse of dimensionality [32,33]. Conversely, ensemble models based on decision trees are structurally simple and perform well in both classification and regression tasks [34]. Random forest (RF) and gradient boosting decision tree (GBDT) exemplify prominent ensemble-based decision tree algorithms that effectively augment nonlinear modeling capacity. Specifically, RF reduces the variance of individual learners through bagging and random feature selection mechanisms, thereby improving overall prediction reliability [35]. Furthermore, research by Xiong et al. [36] demonstrates that RF can effectively handle spatial heterogeneity and characterize complex local features in soil salinization inversion. GBDT is an advanced ensemble decision tree algorithm. It achieves precise modeling of intricate nonlinear relationships by iteratively fitting residuals and aggregating multiple weak learners to gradually optimize the objective function [37,38]. Existing research indicates that, compared with traditional approaches such as linear regression and SVM, GBDT significantly improves prediction accuracy. In addition, DL models further enhance modeling efficiency and precision through automated feature extraction [39–41]. However, most DL algorithms originate from deep neural networks. Despite exhibiting powerful functionality, they entail numerous adjustable parameters, are sensitive to hyperparameter settings, and require substantial data and computational resources, limiting their applicability to datasets with small sample sizes [42,43]. To address these limitations, Zhou et al. [44] proposed the deep forest (DF) model, which integrates the hierarchical feature representation capabilities of DL with the structural characteristics of decision trees. By incorporating a “deep” cascading architecture, DF enhances complex data representation while retaining the greedy segmentation mechanism of decision trees, thereby achieving a balance between model expressiveness and computational efficiency. Zhang et al. [45] employed the DF algorithm to develop a soil cadmium (Cd) content prediction model, achieving a coefficient of determination (*R*^2^) of 0.873, root mean square error (RMSE) of 0.120 mg·kg^-1^, and residual prediction deviation (RPD) of 2.892 on the test dataset, indicating high predictive accuracy and robustness. According to Yang et al. [46], the DF model achieved 93% average accuracy in identifying single-gene deletions, surpassing conventional algorithms such as SVM and k-nearest neighbor (KNN). Bao et al. [47] applied the DF model to precipitation inversion and observed higher predictive accuracy compared to other ML algorithms. However, its application in SSC estimation remains underexplored. Therefore, this study compares the DF model, as an ensemble learning approach, with representative ML algorithms, including SVM, RF, and GBDT, aiming to provide methodological support for the high-precision estimation of SSC.

This study investigates soils within the tilling layer of the Weigan-Kuqa River Oasis in Xinjiang, China. This region represents a typical case of oasis salinization in arid regions, characterized by large-scale salt deposition, widespread salinization, and pronounced salinization-related ecological features. As a representative study area for soil salinization inversion and remote sensing monitoring in arid zones, current research faces challenges including complex spectral response mechanisms, insufficient methodological diversity, and limited model applicability. Therefore, establishing a systematic and robust monitoring framework for soil salinization in arid regions is urgently needed. This study introduces a novel integrated framework that combines multispectral transformations with multi-model algorithms and systematically evaluates its applicability for estimating soil salinity content (SSC). It elucidates the complementarity of different spectral transformation methods and their synergistic advantages when combined with models. The research objectives are as follows: (a) Systematically evaluate the effects of different spectral transformations on enhancing spectral sensitivity, explore the complementarity of their combined use, and identify the optimal spectral characteristic bands; (b) Assess the prediction accuracy and stability of different models to determine the optimal combination of spectral transformations and models for accurate estimation of soil salinization levels; (c) Propose a methodological framework for monitoring soil salinization in oases of arid areas, providing scientific basis for land degradation management.

## Materials and methods

### Research area

The Weigan-Kuqa River Oasis (82°05′ E–83°46′ E, 40°58′ N–41°51′ N) is situated on the southern foothills of the Tianshan Mountains in Xinjiang, China [48]. It encompasses the cities of Kuqa, Xinhe County, and Xayar County within the Aksu Prefecture. The overall terrain of this oasis is relatively flat, gradually transitioning from the Tianshan Mountains in the northwest to the alluvial plains in the central, ultimately extending into the desert region in the south. This creates a distinct topographic gradient sloping from northwest to southeast. Major crops cultivated in this area include jujube (*Zizyphus jujube* *Mill*.), cotton (*Gossypium hirsutum L.*), wheat (*Triticum aestivum L.*), corn (*Zea mays L.*), and walnut (*Juglans regia L.*). Simultaneously, desert vegetation such as Tamarisk (*Tamarix taklamakanensis*), camel thorn (*Alhagi spp.*), Halocnemum strobilaceum (*Halocnemum strobilaceum (Pall.) M. Bieb.*), and Karelinia capsica (*Karelinia capsica (Pall.) Less.*) is widely distributed. The Weigan River, Kuqa River, and Tarim Rivers serve as the primary water sources for the region. Due to low-lying terrain, shallow groundwater levels, intense evaporation, and sparse precipitation, soil salinization is a prominent issue here. The area also features diverse soil types, primarily including brown desert soils, irrigated clay soils, marsh soils, and saline-alkali soils.

### Collecting soil samples and measuring spectrometry

**Fig 1:**
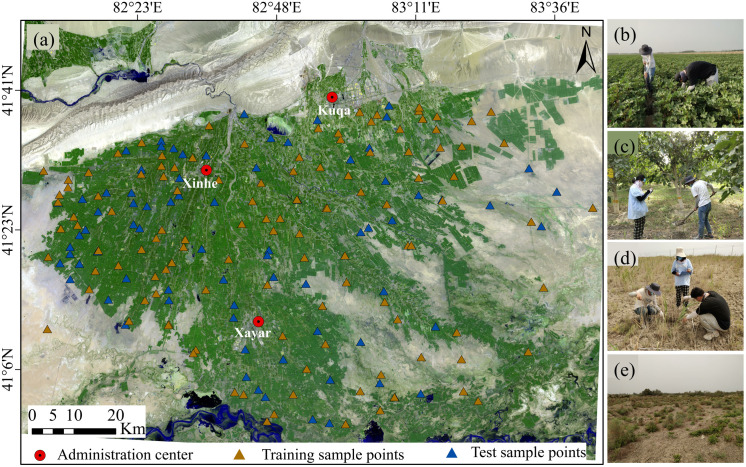
Spatial distribution map of sampling points in the study area and landscape photos. **(a) Spatial distribution of sampling points; (b) Arable land; (c) Garden land; (d–e) Unused land.** Note: The Sentinel-2 image used in Fig 1a was obtained from the Copernicus Data Space Ecosystem website (https://browser.dataspace.copernicus.eu/). The sample point data were derived from field surveys, and the county-level point data were determined based on the GPS coordinates of the county centers. Figs 1b-e are field photos taken by Professor Xuemei Wang at representative sampling sites in the Weigan-Kuqa River Oasis in 2022.


A total of 193 sample points were randomly set up in this study, with their spatial distribution taking into account differences in land use types, soil salinization degree, and surface conditions (Fig 1). Field sampling was conducted in July 2019 and July 2022 (with 98 and 95 samples respectively), during which the cumulative degree of surface soil salinization was the most significant. The sample points covered arable land (64.25%), garden land (15.54%), and unused land dominated by desert shrubbery (20.21%). Tillage layer soil (0–20 cm) was sampled after removing plant roots, stones, and other debris. The collected samples were thoroughly homogenized, sealed in labeled bags, and accompanied by recorded GPS coordinates and site conditions. Subsequently, the soil samples were transported to the laboratory for natural air drying and then ground through a 2 mm standard sieve. After grinding, each soil sample was divided into two aliquots, one for hyperspectral measurement and the other for SSC determination. The portion used for hyperspectral measurement was collected using an ASD FieldSpec 3 portable ground-object spectroradiometer under cloudless outdoor conditions. Dark current correction and whiteboard calibration were performed before each measurement, recording spectral data in the 350–2500 nm range. Each sample underwent 10 spectral measurements, with the average value calculated as the raw spectral reflectance. The portion used for SSC determination was processed following standard electrical conductivity analysis procedures. Soil extracts were prepared at a water-to-soil ratio of 5:1, and the electrical conductivity (EC) of the extracts was measured using an electrical conductivity meter. The measured EC values were then converted into SSC based on an established calibration equation:

SSC=0.0035×EC+0.8570 (1)

Where: EC is the electrical conductivity of the soil sample, in μS·cm ⁻ ¹; SSC indicates the soil salt content, with units of g·kg ⁻ ¹.

### Data preprocessing and spectral transformation

To enhance the accuracy of the spectral data and reduce background and instrument noise interference, spectral-end data in the 2451–2500 nm range, along with bands strongly affected by water absorption in the 1341–1400 nm and 1811–1950 nm regions, were excluded. Ultimately, 1901 wavelengths spanning from 350 to 2450 nm were selected for subsequent analysis. The original spectral reflectance was first smoothed using the Savitzky-Golay method to suppress noise. Building upon the foundation of our research team’s prior studies and extensive preliminary experiments, we further screened three transformation methods from twelve mathematical transformations that effectively enhance the correlation between spectral data and soil salinity. These three methods, applied to the smoothed spectral reflectance (*R*), are the first-order differential of reciprocal (1/*R*)′, the first-order differential of logarithmic (lg*R*)′, and the first-order differential of the logarithmic reciprocal [lg(1/*R*)]′ [49].

Continuous wavelet transformation (CWT) is a time-frequency analysis method characterized by high resolution and adaptability. It is highly effective in suppressing spectral noise and extracting weak signals. It convolves reflectance data with scaled and translated wavelet functions to decompose spectral reflectance at different scales, generating a series of wavelet coefficients [50]. The computation formula is presented below [51]:


$$\omega_{ij}={\int_{-\infty }^{+\infty }\nu_{ij}\psi_{a,b}(j)d}_{j}$$ (2)

In this formula, $\omega_{ij}$ and $v_{ij}$ represent the wavelet coefficient and reflectance corresponding to the *j*th band of the *i*th soil sample, respectively. Additionally, *a* and *b* denote the scale and translation factors, respectively. While $\psi_{a,b}(j)$ represents the wavelet basis function.


$$\psi_{a,b}(j)=\frac{\text{1}}{\sqrt{a}}\psi \left(\frac{j-b}{a}\right)$$ (3)

Based on the existing research [29], the bior1.3 wavelet basis function was employed to decompose the spectrum across 10 scales, denoted as 2^1^, 2^2^, 2^3^, 2^4^, 2^5^, 2^6^, 2^7^, 2^8^, 2^9^, and 2^10^.

Discrete wavelet transformation (DWT), a powerful signal processing technique, decomposes signals into low-frequency (approximation) and high-frequency (detail) coefficients across multiple scales via wavelet functions [52]. Through this multiscale decomposition, it can effectively eliminate unwanted noise components while extracting valuable information from different frequency bands [53]. The decomposition formula can be expressed as below [54]:


$$f(\lambda )=a_{j}(\lambda )+\sum_{i=\text{1}}^{j}d_{i}(\lambda )$$ (4)

In the equation, f(λ) signifies the spectral signal, *j* denotes the decomposition layer, $a_{j}$ corresponds to the low-frequency component, and $d_{i}$ indicates the high-frequency component. Based on the existing research [30], by comparing the commonly used wavelet basis functions such as Haar, db4, db5, sym2, and Bior1.3, this study finally adopts the sym2 wavelet which has the highest correlation with SSC. The spectral data is decomposed into 10 layers using the wavelet method, and the approximate components and detail components of each layer are sequentially labeled as L1 to L10 and H1 to H10.

### Feature band screening method

Spectral characteristic bands were identified using the Pearson correlation coefficient (*r*). This coefficient quantifies the linear relationship between variables and is commonly employed to evaluate the association between SSC and spectral reflectance. The value of *r* ranges from −1–1, where values approaching 1 indicate a strong positive correlation and those near −1 suggest a strong negative correlation. The computational formula for *r* is given below [55]:


$$r_{xy}=\frac{cov(X,Y)}{\sigma_{x}\sigma_{y}}=\frac{\sum_{i=\text{1}}^{n}\left(x_{i}-\overset{―}{x}\right)\left(y_{i}-\overset{―}{y}\right)}{\sqrt{\sum_{i=\text{1}}^{n}{\left(x_{i}-\overset{―}{x}\right)}^{\text{2}}}\sqrt{\sum_{i=\text{1}}^{n}{\left(y_{i}-\overset{―}{y}\right)}^{\text{2}}}}$$ (5)

Here, *n* is defined as the sample size; $x_{i}$ represents the measured SSC at point *i*, and $\overset{―}{x}$ is the mean SSC across all points. $y_{i}$ corresponds to the reflectance at sample *i*, and $\overset{―}{y}$ is the mean reflectance across all points.

### Principles of the estimation model

Random forest (RF) is an ensemble algorithm that constructs multiple independent decision trees by randomly selecting samples and features. The final prediction is obtained by aggregating the outputs of individual trees using either a voting method or an averaging method [56]. RF exhibits strong noise resistance and fitting ability, and performs well on both large-scale and limited datasets [57]. Gradient boosting decision tree (GBDT) is an ML algorithm that integrates decision trees with boosting serial ensemble learning strategies. The algorithm gradually optimizes model performance through multiple rounds of iteration, and its core concept involves fusing multiple weak learners into a strong predictor through weighted aggregation [58,59]. Support vector machine (SVM) is founded on the principle of structural risk minimization. It maps the original data into a high-dimensional feature space using kernel functions, thereby converting complex nonlinear regression problems into linearly solvable ones [60]. SVM is known for its strong learning capacity and excellent generalization performance in small-sample scenarios, making it particularly suitable for high-dimensional nonlinear modeling tasks [61]. Deep forest (DF) is a non-neural-network deep learning algorithm based on ensemble decision trees. It performs feature extraction through multi-granularity scanning and a cascaded forest structure [62]. DF combines RF and stacking strategies within a layer-wise architecture analogous to deep neural networks. It offers both powerful feature representation and robustness in small-sample conditions, and its adaptive depth adjustment mechanism effectively combats overfitting, rendering it suitable for high-dimensional small-sample modeling [63]. The parameter settings for each model are shown in Table 1.

**Table 1:** The values of each model parameter(s).

model	model parameter	(1/*R*)′	*R*-CWT-2^7^	(1/*R*)′-CWT-2^7^	*R*-DWT-H7	(1/*R*)′-DWT-H8
SVM	kernel function	rbf	rbf	rbf	rbf	rbf
	C	3.9	12	2	1	1
	γ	8.1	14	5.8	11.12	5.94
RF	trees	12	6	5	4	6
	leaf	7	3	2	4	3
GBDT	num_trees	100	100	100	500	100
	learning_rate	0.027	0.35	0.053	0.064	0.074
	max_depth	3	3	3	3	3
	subsample	0.25	0.25	0.81	0.555	0.435
	min_leaf_size	5	5	5	5	5
	min_impurity_decrease	0.01	0.01	0.01	0.01	0.01
	test_ratio	0.4	0.4	0.4	0.4	0.4
	patience	5	5	13	5	12
DF	window_size	$\frac{\text{1}}{\text{8}}x\;$（， $\frac{\text{1}}{\text{3}}x$）， *x* represents the number of features, with a step size of 1				
	n_estimators	5	2	9	2	3
	n_trees	16	17	23	15	17
	max_layers	5	2	1	3	7
	min_samples_split	4	3	4	2	3
	min_samples_leaf	3	2	3	2	3
	random_state	24	24	46	24	24

### Methods for verifying the accuracy of the model

This study employed the coefficient of determination (*R*^2^), root mean square error (RMSE), and residual prediction deviation (RPD) to assess the performance of the SSC estimation model. The *R*^2^ value ranges from 0 to 1, with higher values indicating a better model fit. A lower RMSE corresponds to higher predictive accuracy. RPD is commonly employed to assess model robustness and predictive capability. Specifically, when the RPD value is below 1.4, it suggests weak predictive performance; an RPD value above 1.4 signifies that the model possesses basic predictive capability, with higher values indicating stronger predictive performance [2]. The corresponding calculation formulas for each evaluation metric are provided below:


$$R^{\text{2}}=\text{1}-\frac{\sum_{i=\text{1}}^{n}{\left(y_{i}-{\hat{y}}_{i}\right)}^{\text{2}}}{\sum_{i=\text{1}}^{n}{\left(y_{i}-\bar{y}\right)}^{\text{2}}}$$ (6)

$$RMSE=\sqrt{\frac{\sum_{i=\text{1}}^{n}{\left(y_{i}-{\hat{y}}_{i}\right)}^{\text{2}}}{n}}$$ (7)

$$RPD=\frac{\sqrt{\frac{\text{1}}{n-\text{1}}\sum_{i=\text{1}}^{n}{\left(y_{i}-\bar{y}\right)}^{\text{2}}}}{RMSE}$$ (8)

Here, *n* denotes the number of samples; ${\hat{y}}_{i}$ is the predicted SSC value for the *i*th sample, and $y_{i}$ is the corresponding observed SSC value. $\overset{―}{y}$ represents the mean of all observed SSC values.

To quantitatively evaluate the differences in prediction performance of the DF model compared to the contrast models (SVM, RF, and GBDT) on the test set, a relative change percentage of the evaluation metric (∆*M)* is introduced. Its calculation formula is as follows:


$$\Delta M(\%)=\frac{M_{DF}-M_{ref}}{M_{ref}}\times \text{1}\text{0}\text{0}\%$$ (9)

Here, *M* denotes the model evaluation metrics (including *R*², RMSE, and RPD), while *M*_*DF*_ and *M*_*ref*_ represent the corresponding metric values achieved by the DF model and the reference model (SVM, RF, or GBDT model) on the test set, respectively.

**Fig 2:**
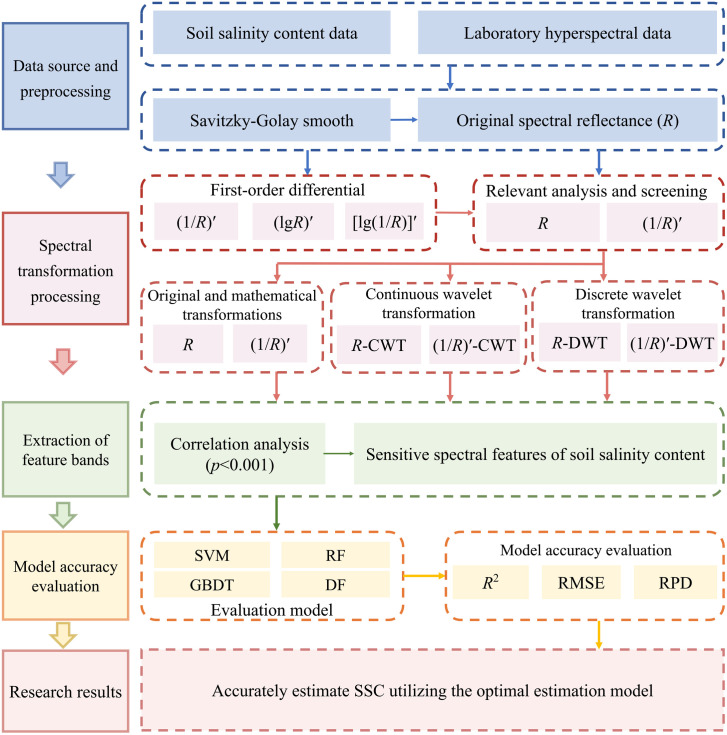
Technical route.

To clearly illustrate the research approach and implementation steps, the technical route of this study is shown in Fig 2.

This research was conducted based on the soil samples collected in the field. All the analytical data (including soil reflectivity and laboratory-determined salt content) are direct measurement results of the physical and chemical properties of the samples, and do not contain any personal identification information, nor involve human participants or animal experiments. Therefore, the research plan was submitted to the Academic Committee of the College of Geographic Sciences and Tourism, Xinjiang Normal University, and was approved for exemption from formal ethical review in accordance with relevant research ethics regulations; meanwhile, the field sampling activities strictly followed the management norms of the relevant locations.

## Results and analysis

### Statistical and spectral feature analysis of SSC

**Fig 3:**
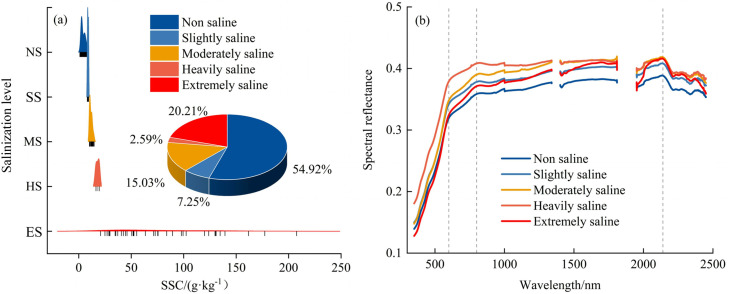
Sample point proportions, salt distribution, and spectral characteristics of different salinization grades.

The average pH value of the soil in the study area is 7.96, indicating that the soil in the Weigan-Kuqa River Oasis is alkaline. Based on the analysis of the contents of eight major ions, the salt in this area is mainly composed of chlorides and sulfates, with a small proportion of chloride-sulfates [64]. According to the salt-alkali classification criteria formulated in the Second Soil Survey of Xinjiang [65], 193 soil samples were divided into five salinity categories: non-saline (SSC < 8 g·kg^-1^), slightly saline (8 ≤ SSC < 10 g·kg^-1^), moderately saline (10 ≤ SSC < 15 g·kg^-1^), heavily saline (15 ≤ SSC < 20 g·kg^-1^), and extremely saline (SSC ≥ 20 g·kg^-1^). As illustrated in Fig 3a, SSC values ranged from 1.084 to 207.615 g·kg^-1^, indicating a wide variation in soil salinity levels across the study area. The mean SSC was 20.800 g·kg^-1^, indicating a generally high level of salinity. Regarding the distribution of salinity grades, non-saline soils accounted for the largest proportion (54.92%), followed by extremely saline soils, while heavily saline soils comprised the smallest share (2.59%). This distribution implies that, although most soils in the study area contain relatively low levels of salt, certain locations exhibit severe salt buildup. This may result from natural factors such as groundwater evaporation and low-lying terrain, combined with anthropogenic factors including improper irrigation and inadequate drainage management. Based on the SSC groupings shown in Fig 3a, the samples were classified into five types, and their average spectral reflectance profiles are depicted in Fig 3b. Although the overall shape of the reflectance curves is similar across all SSC grades, distinct differences are observed at specific wavelengths, indicating that SSC influences soil spectral properties. In the visible range of 350–600 nm, reflectance increases sharply with wavelength, showing minimal differences between salinity levels. From 600 to 800 nm, reflectance continues to rise gradually, and inter-class differences become more pronounced. Between 800 and 2140 nm, reflectance stabilizes and peaks near 2140 nm, after which it declines. Differences among the salinity grades gradually decrease in this region. Notably, the absorption feature near 2215 nm is attributed to the vibrations of Al–OH groups in clay minerals, while the absorption feature near 2335 nm is associated with the vibrations of CO₃²⁻ in carbonate minerals [66]. From the overall perspective of the spectral curve, the range of soil spectral reflectance is 0.128 to 0.420. Reflectance generally increases with SSC from non-saline to heavily saline soils, possibly due to enhanced scattering by surface salt crystals. However, at extremely saline levels, the reflectance decreases, mainly because salt has strong absorption properties at specific wavelengths, causing the soil to absorb rather than reflect the spectrum in that wavelength range, especially more pronounced in the 350–1500 nm range [2]. This nonlinear pattern reflects the complex interplay between salinity, surface properties, moisture, and spectral performance in arid oasis soils.

### Correlation analysis and feature extraction under different spectral processing methods

**Fig 4:**
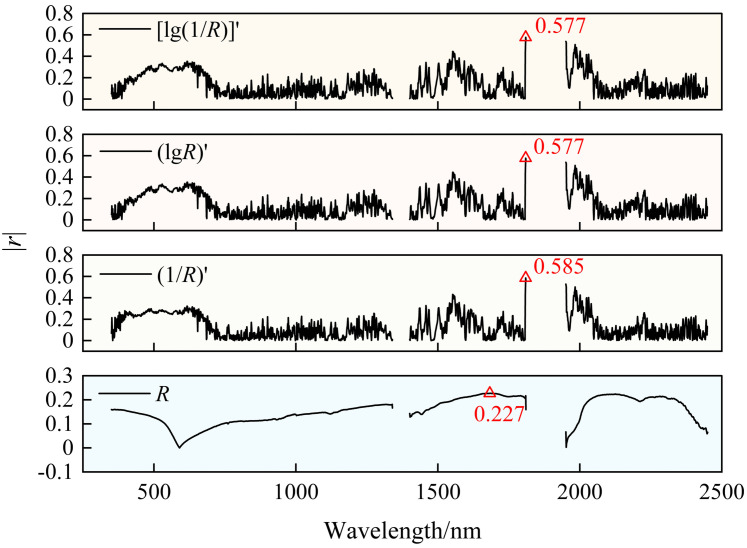
Correlation between mathematically transformed Spectra and SSC.

Correlation analysis between various spectral transformations and SSC facilitates the identification of spectral bands most sensitive to SSC variations. As illustrated in Fig 4, the correlation between *R* and SSC is relatively weak, with the maximum correlation coefficient (*r*_max_) being only 0.227 (1684 nm). This mainly arises from spectral nonlinearities caused by the interaction of soil background noise, water absorption, and salt content, which hinder the original reflectance from accurately capturing the spectral responses to salt content variations. To enhance the responsiveness of spectral signals to SSC changes, three mathematical transformations — (1/*R*)′, (lg*R*)′, and [lg(1/*R*)]′ — were applied to *R*. These transformations amplify subtle fluctuations and enhance local slope changes by adjusting the morphology of the spectral curves, thereby helping to highlight characteristic information in the original spectra that is weakened or masked by background interference or water absorption effects. The correlation between the transformed spectra and SSC was significantly improved. All three showed the strongest correlation at 1809 nm, with the absolute values of the *r* (|*r*|) being 0.585, 0.577, and 0.577, respectively. Among these, the (1/*R*)′ transformation further highlights the weak influence of salt on the spectral curve by compressing the high-reflection region and enhancing the fluctuations in the low-reflection region, thereby showing the best correlation among the three transformations. Consequently, this transformation was selected for the subsequent wavelet decomposition and feature band extraction.

**Fig 5:**
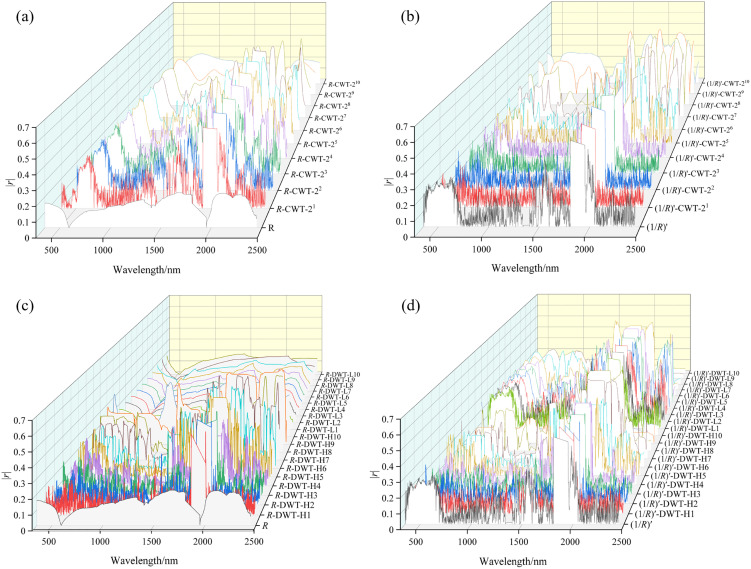
Correlation between wavelet coefficients at various scales and SSC under different wavelet transformations.

To investigate the effect of wavelet transformation on improving spectral sensitivity, both CWT and DWT were applied to the *R* and its transformed form (1/*R*)′, generating four datasets: *R*-CWT, (1/*R*)′-CWT, *R*-DWT, and (1/*R*)′-DWT. Subsequently, the |*r*| between wavelet coefficients and SSC was calculated (Fig 5). The results indicate that the wavelet transformation improves the correlation between spectral features and SSC compared to *R*. In CWT, the |*r*|_max_ values at each scale for *R*-CWT and (1/*R*)′-CWT both show a trend of first increasing and then decreasing as the decomposition scale increases. Both reach their peaks at the 2^7^th scale, with the corresponding |*r*| values being 0.592 (2041 nm) and 0.598 (1991 nm), respectively. This indicates that decomposition at medium and high scales can effectively suppress high-frequency noise while retaining SSC information, with particularly strong enhancement of weak absorption features in the near-infrared band. For DWT, the low-frequency scale wavelet coefficients of *R*-DWT have a generally weak correlation with SSC, with |*r*| values below 0.242. The |*r*|_max_ values of the high-frequency scales first increases and then decreases with increasing decomposition scale, reaching a peak at the H7 scale (0.621, 1980 nm). This indicates that the high-frequency scale is more conducive to extracting detailed SSC-related information, but too small a scale is prone to noise interference, while too large a scale makes the signal overly smooth—both reducing the correlation. In contrast, (1/*R*)′-DWT shows strong correlations in multiple high-frequency and low-frequency scales. The |*r*|_max_ values from H1 to H8 and L1 to L7 are all greater than 0.494, with the highest value (|*r*|_max_ = 0.603) at the H8 scale and the lowest value (|*r*|_max_ = 0.203) at the L10 scale. This indicates that (1/*R*)′, by enhancing spectral slope changes and highlighting weak absorption features, enables the extraction of effective SSC information across multiple high- and low-frequency DWT scales. These findings indicate that both CWT and DWT can enhance subtle signals. Furthermore, combining mathematical transformations with CWT and DWT can further enhance the weak signals and help extract the feature information highly relevant to SSC.

**Fig 6:**
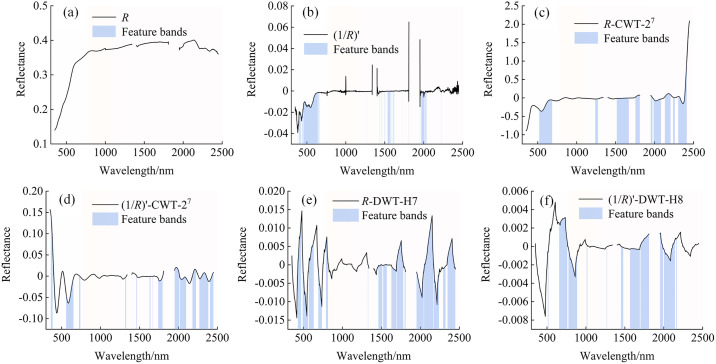
Feature band distributions under various spectral transformations.

Feature band selection helps reduce model complexity and enhance prediction robustness. In the previous study [49], the correlations between significant bands (*p* < 0.01) and SSC, as well as the modeling effects, were systematically analyzed. Based on this previous study, this research further calculated the correlations between extremely significant bands and SSC, and compared them with the previous study results. It was found that although the number of extremely significant bands was small, their correlations with SSC were more significant. Therefore, based on the correlation analysis results, this research selected the bands highly significant correlated (*p* < 0.001, |*r*| > 0.235) with SSC from the optimal mathematically transformed spectrum ((1/*R*)′) and the optimal decomposition scales of wavelet transformations as the feature bands. Fig 6 illustrates the distribution of feature bands extracted from different spectral transformations. *R* (Fig 6a) failed to extract any feature bands, primarily due to prominent background noise and environmental interference during spectral acquisition, which masked the faint spectral signals associated with SSC. In contrast, (1/*R*)′ (Fig 6b) enhanced the changes at the edge of the absorption band and suppressed background interference, thereby improving the recognition ability of spectral features and extracting 396 characteristic bands—mainly distributed in the visible light (456–672 nm) and short-wave infrared regions (1523–1579 nm, 1808–1955 nm, and 1978–2014 nm). Fig 6c and 6d show that 740 and 577 discontinuously distributed feature bands were extracted from the 2^7^thscale of the *R*-CWT (*R*-CWT-2^7^) and the 2^7^th scale of the (1/*R*)′-CWT ((1/*R*)′-CWT-2^7^), respectively. These feature bands exhibit a continuous distribution within the visible light range of 558–652 nm, while in the shortwave infrared range of 1500–2400 nm, they are primarily distributed across multiple discrete intervals. This is primarily because CWT enhances spectral local details through multiscale continuous decomposition, making it easier to identify weak spectral features. In contrast, DWT employs hierarchical discrete decomposition, which effectively preserves the main spectral structure and key absorption features. Fig 6e and 6f show that the H7 scale of the *R*-DWT spectrum (*R*-DWT-H7) and the H8 scale of the (1/*R*)′-DWT spectrum ((1/*R*)′-DWT-H8) generated 929 and 700 characteristic bands, respectively, covering a broader range. Specifically, the feature bands of *R*-DWT-H7 span nearly the entire spectrum, while those of (1/*R*)′-DWT-H8 exhibit higher correlation (|*r*| > 0.602) in the 1969–1971 nm and 2110–2114 nm ranges. This indicates that DWT holds advantages in preserving overall spectral trends and key features, enabling the extraction of more representative SSC-sensitive bands. Despite variations in the number and distribution of selected bands across different spectral transformations, some consistent SSC-sensitive bands were observed. These include the clay mineral absorption band near 1633 nm, hydrated ion absorption bands at 1809–1810 nm and 1951–1955 nm, crystalline water and hydroxyl absorption bands at 1969–1971 nm and 1987–1989 nm, as well as several weak absorption bands related to soluble salts in the ranges 2001–2012 nm, 2021–2026 nm, 2029–2034 nm, and 2038–2041 nm. These bands are mostly associated with negative ions such as OH⁻ and CO₃²⁻ and mineral absorption properties. Salt accumulation may enhance spectral responses by altering the mineral structure and hydration state.

### Sample set division

**Fig 7:**
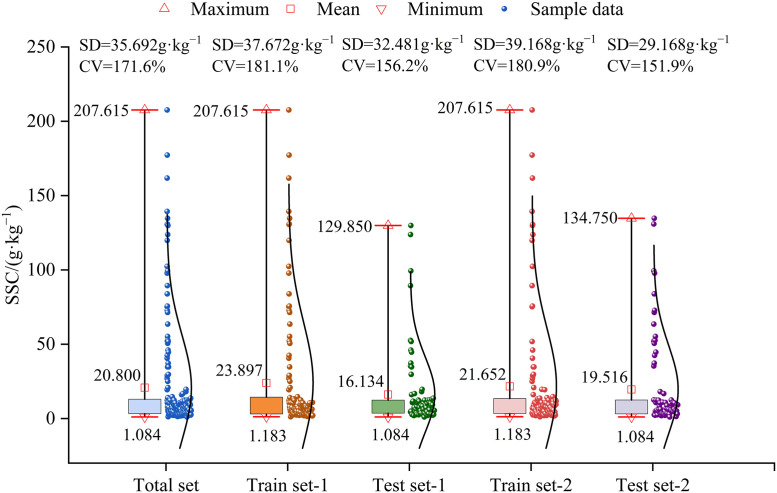
Descriptive statistics of SSC in each sample set.

A scientifically sound division of the sample set is a crucial prerequisite for ensuring model accuracy and stability. In this study, 193 samples were randomly partitioned into a training set (116 samples) and a test set (77 samples) at a 6:4 ratio. As shown in Fig 7, the SSC values of the total sample set ranged from 1.084 to 207.615 g·kg^-1^ and exhibited high dispersion, with a mean of 20.800 g·kg^-1^—indicating a generally high level of soil salinization. The coefficient of variation (CV) was 171.6%, reflecting the significant spatial heterogeneity of salinization in the study area. To verify the representativeness of the sample partitioning, the means, standard deviations (SD), and CV of both the training and test sets were analyzed. The data underwent two independent random partitions, with statistical characteristics calculated separately for each set. Results showed that the mean and SD of the training and test sets obtained from both divisions closely matched those of the total sample set, with the training set being slightly higher and the test set slightly lower. The CV exceeded 151.9% for both sets, indicating that the divided subsets effectively reflected the distribution and variability of the overall sample set. These findings confirmed the representativeness of random division, providing reliable and robust support for subsequent model analysis.

### Model establishment and accuracy evaluation

**Fig 8:**
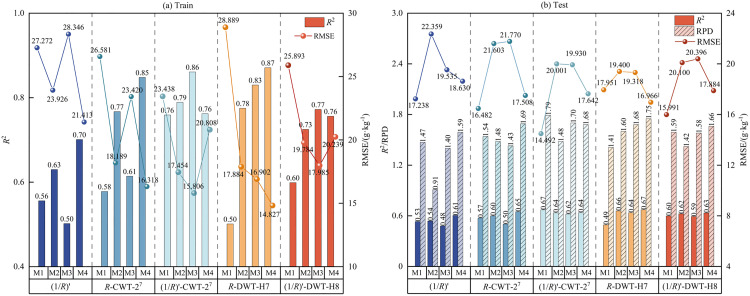
Accuracy metrics of the SSC estimation model in training and testing Phases. Note: M1 corresponds to the SVM; M2 to the RF; M3 to the GBDT; M4 to the DF.

To evaluate the effects of different spectral transformations on SSC prediction, bands highly significantly correlated with SSC (*p* < 0.001, |*r*| > 0.235) were selected as input variables to construct models based on the RF, SVM, GBDT, and DF algorithms. The results are presented in Fig 8. Among the five transformations, the (1/*R*)′-CWT-2^7^ transformation exhibited the best performance. In the training set, *R*^2^ values exceeded 0.75, and RMSE values remained below 23.438 g·kg^-1^. In the test set, *R*^2^ values ranged from 0.62 to 0.67, and RPD values all exceeded 1.48, demonstrating robust predictive performance. The *R*-DWT-H7 transformation also showed stable performance, particularly in ensemble models (RF, GBDT, DF), where training *R*^2^ values exceeded 0.78 and test *R*^2^ values ranged from 0.64 to 0.67. In contrast, the SVM model performed poorly, reducing the overall stability of the predictions. Models utilizing the (1/*R*)′-DWT-H8 transformation produced moderately stable results, with test *R*^2^ values ranging from 0.59 to 0.63. The *R*-CWT-2^7^ transformation exhibited higher variability in training but maintained consistent test performance (*R*^2^ ≈ 0.57, RPD > 1.43). Models based solely on (1/*R*)′ achieved the lowest accuracy, with training *R*^2^ values generally below 0.63 and test *R*^2^ values between 0.480 and 0.61, indicating suboptimal model fitting and limited predictive ability. Overall, combining mathematical transformation with CWT achieved the most favorable modeling results, followed by DWT, mathematical transformation combined with DWT, and CWT alone. Models relying solely on mathematical transformation exhibited the poorest performance, confirming that wavelet transformations—especially when combined with mathematical transformation, can effectively improve model accuracy and stability. From the performance of different models under the same spectral transformations, in the (1/*R*)′, *R*-CWT-2^7^, and *R*-DWT-H7 transformations, the DF model achieved relatively high *R*^2^ values on both the training and test sets, indicating that it has relatively strong and stable modeling ability. Under the (1/*R*)′-DWT-H8 transformation, except for the SVM model, which had relatively weak fitting ability on the training set, the other models performed well. Among them, the DF model achieved the highest *R*^2^ value (0.63) and a relatively lower RMSE value (17.884 g·kg^-1^) on the test set, with the best comprehensive performance. Overall, except for the (1/*R*)′-CWT-2^7^ transformation, the DF model performed the best under most spectral transformation conditions.

**Fig 9:**
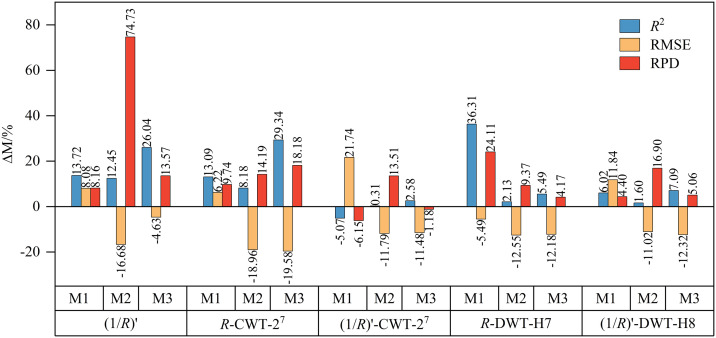
The relative percentage change in performance of the DF model compared to SVM, RF and GBDT on the test set.

To assess the degree of accuracy improvement of the DF model compared to the SVM, RF, and GBDT models, the relative performance change percentage (Δ*M*) of the DF model in each evaluation indicator was calculated based on the *R*², RMSE, and RPD values of the SVM, RF, and GBDT models on the test set (Fig 9). The results show that the DF model outperforms the other models under most spectral transformations, particularly in the *R*-DWT-H7 processing. It exhibits significant improvements in *R*^2^, RMSE, and RPD, showcasing its advantages in predictive accuracy, stability, and generalization capability. In the (1/*R*)′ and *R*-CWT-2^7^ transformations, the DF model achieved higher *R*^2^ and RPD values than all three comparative models. Although its RMSE was marginally higher than that of SVM, it showed notable reductions compared to RF and GBDT, with the largest decrease reaching 19.58%, indicating that it has strong predictive ability and generalization performance overall. Under the (1/*R*)′-CWT-2^7^ transformation, the DF model outperformed RF in all three metrics and outperformed GBDT in *R*^2^ and RMSE. While the SVM model was optimal in all three evaluation indices under this transformation, indicating that it also has strong modeling ability and generalization performance in specific spectral transformations. Under the (1/*R*)′-DWT-H8 transformation, the DF model achieved a higher *R*^2^ than the SVM, RF, and GBDT models. Although its RMSE was 11.84% higher than that of SVM, it was significantly lower than those of the RF and GBDT models. Additionally, its RPD value improved compared to both the SVM and RF models. In summary, the DF model demonstrates superior prediction performance across most spectral transformations, particularly in (1/*R*)′, *R*-CWT-2^7^, and *R*-DWT-H7 processing, where its accuracy is significantly enhanced, highlighting its adaptability and application potential in SSC prediction.

**Fig 10:**
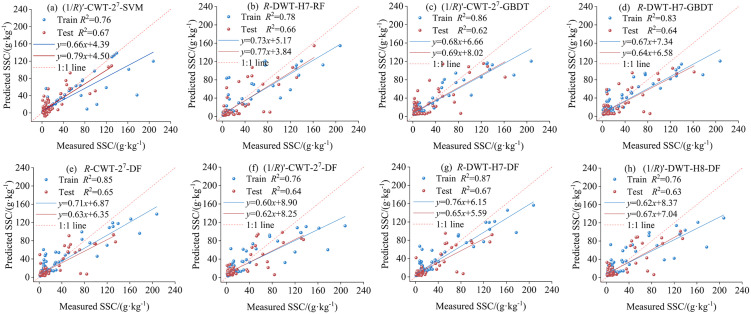
Scatter plot of estimated vs. measured SSC values.

To assess the predictive accuracy of SSC under various spectral transformation and modeling combinations, based on the evaluation of the prediction results of all models on the training set and the test set, only the scatter plots of observed values versus predicted values were drawn for the models with better predictive performance (RPD > 1.60) on the test set (Fig 10). The results reveal considerable variation in predictive capabilities among the various combinations. Among these, the *R*-DWT-H7-DF model (Fig 10g) performed optimally, achieving *R*^2^ values of 0.87 and 0.67 for the training and test sets, with corresponding fitting slopes of 0.76 and 0.65. The data points aligned relatively well with the fitted line, indicating that the model possesses relatively good accuracy and stability. The (1/*R*)′-CWT-2^7^-SVM model (Fig 10a) ranked second in performance, yielding *R*^2^ values of 0.76 and 0.67 for the training and test sets. The corresponding fitting slopes of 0.66 and 0.79 were both close to the 1:1 reference line, indicating favorable predictive accuracy. The *R*-DWT-H7-RF model in Fig 10b also showed strong performance, yielding training and test set *R*^2^ values of 0.78 and 0.66, with fitting slopes of 0.73 and 0.77, respectively. While other combinations showed training set *R*^2^ values ranging from 0.76 to 0.86, their test set *R*^2^ values were generally below 0.65, and the slopes deviated from the ideal line, indicating lower prediction accuracy. In particular, the (1/*R*)′-CWT-2^7^-GBDT model (Fig 10c) had a high training *R*^2^ of 0.86, but the test *R*^2^ dropped to 0.62, reflecting limited generalizability. In conclusion, the choice of spectral transformation and modeling approach greatly influences SSC prediction accuracy, with the *R*-DWT-H7-DF model emerging as an effective combination. Thus, this combination can be considered the optimal solution for SSC estimation.

## Discussion

### The influence of spectral transformation on correlation enhancement and modeling accuracy

This study compared the correlations between three FD spectra — (1/*R*)′, (lg*R*)′, and [lg(1/*R*)]′ — and SSC. Results indicate that (1/*R*)′ is the optimal mathematical transformation (Fig 4), consistent with findings by Sun et al. [2]. This is primarily because SSC-sensitive bands often overlap with absorption regions of clay minerals and moisture, resulting in weak spectral features. (1/*R*)′ effectively amplifies these weak salinity-related absorption signals while suppressing overall baseline background interference [49]. However, Xia et al. [67] identified (lg*R*)′ as the optimal FD transformation for SOC estimation, differing from our findings. This discrepancy arises because SOC’s spectral response in the visible-near infrared region primarily stems from harmonic and sum-frequency absorption of functional groups like OH, NH, and CH, typically manifesting as broad, overlapping absorption bands characterized by reduced reflectance [68]. (lg*R*)′ effectively eliminates background noise caused by factors such as soil particle scattering and enhances the slope variation of broad absorption bands, thereby making the broad spectral features associated with SOC more distinct and prominent [69]. Compared with traditional transformations, WT offers greater advantages in feature extraction. Han et al. [27] reported that CWT effectively suppresses spectral noise and enhances feature information. The correlation and modeling accuracy of wavelet coefficients extracted at L3-L5 scales with SOC content were superior to those obtained using the FD transformation. Qi et al. [70] also found that spectra processed using CWT exhibited the highest correlations with protein and wet gluten content. When combined with ReliefF and SVM, the classification accuracy reached 94.5%, significantly outperforming the *R* and FD methods. This study likewise demonstrates that CWT outperforms the FD transformation in improving correlation with SSC and model accuracy (Figs 5 and 8). Furthermore, DWT also performs outstandingly in enhancing spectral correlation and improving modeling accuracy. Both Liu et al. [26] and this study indicate that DWT significantly outperforms both the *R* and FD spectra in enhancing spectral correlation with the target variable and improving modeling accuracy. Research by Roy et al. [71] revealed that CWT outperforms DWT in predicting SOC and mineral content; however, in this study, DWT demonstrated superior predictive performance for SSC. The spectral characteristics of SOC typically exhibit continuous and concentrated distribution patterns, which CWT can effectively capture. In contrast, the sensitive bands for SSC in the shortwave infrared region often manifest as localized absorption troughs and abrupt peaks. Leveraging its hierarchical discrete decomposition properties, DWT can suppress noise while better preserving the overall spectral trends and critical local variation features. This enables more precise extraction of SSC-related sensitive bands, thereby enhancing prediction accuracy [72]. In particular, the *R*-DWT-H7 spectrum exhibited the highest correlation with SSC (|*r|* = 0.621) (Fig 5), consistent with the conclusion of Hang et al. [73], who reported that the *R*-DWT-H9 spectrum significantly enhanced the correlation with SOC content. Both studies highlight the effectiveness of DWT in extracting relevant spectral features. In addition, Liu et al. [74] found that combining DWT with FD can effectively highlight detailed features and suppress noise, yielding better prediction accuracy than using DWT alone. However, in this research, the (1/*R*)′-DWT-H8 model performed worse than the DWT model alone (Fig 8), which may be due to high-frequency noise introduced by (1/*R*)′ being superimposed on the high-frequency coefficients of the selected wavelet during modeling [75]. This study further revealed that combining the differential transform with CWT significantly improved the correlation with SSC, yielding an increase in *r* of 0.371 (Fig 5). The resulting model outperformed those based on either the differential transformation or CWT alone. This finding is consistent with the results reported by Xiao et al. [76].

### Impact of feature band screening on prediction results and analysis of feature band distribution

**Fig 11:**
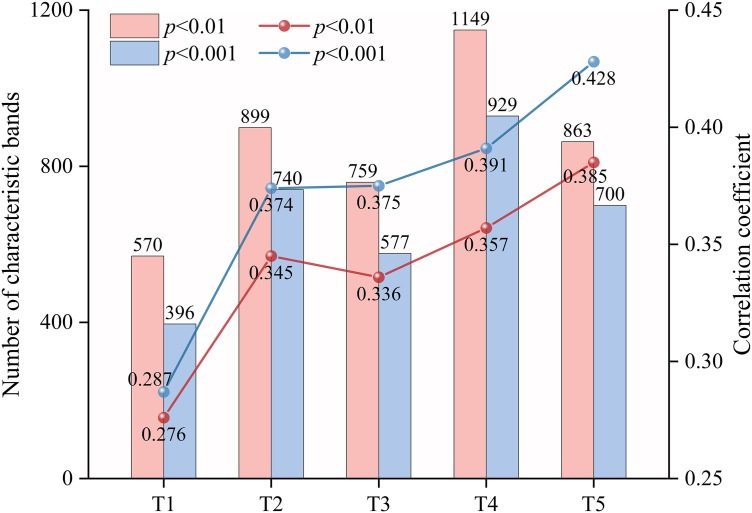
Number of significantly and extremely significantly correlated bands and the mean |*r*|. Note: T1 denotes (1/*R*)′; T2 denotes *R*-CWT-2^7^; T3 denotes (1/*R*)′-CWT-2^7^; T4 denotes *R*-DWT-H7; T5 denotes (1/*R*)′-DWT-H8.

**Fig 12:**
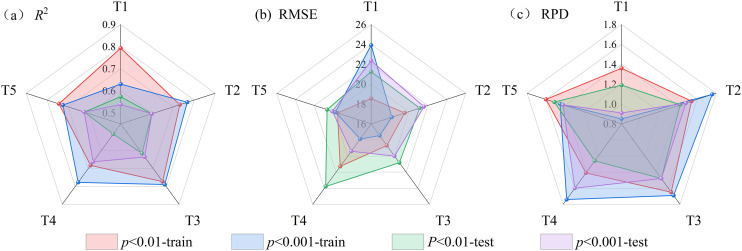
Estimation results of RF models for significantly correlated and extremely significantly correlated bands. Note: T1 denotes (1/*R*)′; T2 denotes *R*-CWT-2^7^; T3 denotes (1/*R*)′-CWT-2^7^; T4 denotes *R*-DWT-H7; T5 denotes (1/*R*)′-DWT-H8.

Feature band selection is crucial for reducing model complexity and improving training efficiency. The Pearson correlation coefficient method enables the extraction of informative spectral features without requiring prior model training [77]. Mao et al. [78] employed both the correlation coefficient method and the Boruta algorithm for band selection. Their results indicated that the number of bands with significant correlations (*p* < 0.01), as determined by the correlation coefficient method, was relatively stable. Moreover, the inversion model for heavy metal content constructed using these bands achieved significantly higher prediction accuracy than that based on Boruta-selected bands. Meanwhile, in the present study, the predictive performance of RF models constructed using significantly correlated bands (*p* < 0.01) and extremely significantly correlated bands (*p* < 0.001) was compared. Although the number of extremely significant bands is relatively small, their average correlation coefficients are significantly higher, indicating that these bands contain more sensitive information regarding soil salinity (Fig 11). Furthermore, the RF model constructed using the extremely significantly correlated bands also outperforms the one based on the significantly correlated bands in predictive performance (Fig 12). This is because the high-correlation bands carry more information about the target variable, which can effectively enhance the discriminative ability of the model and thus more accurately fit the changes in SSC [79]. At the same time, these bands also reduce the redundancy and noise interference in the input features, improving the stability and generalization ability of the model training and ultimately enhancing the overall prediction performance [80]. Previous studies have explored the distribution of SSC-sensitive bands. Wang et al. [56] applied various spectral transformations and employed the SPA method to identify SSC-related bands, identifying those frequently selected across multiple transformations as salt-sensitive. Their results indicated that the spectral ranges of 525–744 nm, 1834–1899 nm, and 1901–2054 nm contained substantial information associated with soil salinity. Similarly, Wang et al. [81] noted that bands within 2000–2200 nm were effective in predicting concentrations of three major salt types under both dry and wet soil conditions. In this study, FD, CWT, DWT, and combinations of FD with CWT or DWT were applied to the *R* to extract bands that were extremely significantly correlated with SSC. Bands consistently identified across multiple transformations were considered salt-sensitive. The final set of extracted SSC feature bands—2001–2012 nm, 2021–2026 nm, 2029–2034 nm, and 2038–2041 nm—fall within the salt-sensitive ranges reported in earlier studies, confirming that the selected bands are indeed indicative of soil salinity.

### Comparison of prediction accuracy based on different models

The findings of this study indicate that, among diverse combinations of spectral transformations and models, the estimation effect of the combination of the FD and RF model is significantly better than that of the GBDT and SVM models (Fig 8). Zhou et al. [82] also demonstrated that the RF model exhibited the best performance by combining multiple mathematical transformations with MLR, PLSR, SVM, and RF algorithms for SOC content assessment. Similarly, the research by Zhou et al. [5] found that FD is the most effective spectral transformation in heavy metal prediction, and the RF model based on FD is significantly superior to the SVR and PLS models. In the study of WT, the results of Roy et al. [71] and Han et al. [27] show that the prediction accuracy of SOC content under CWT treatment reaches its highest. Meanwhile, the RF model exhibits strong robustness in different spectral transformations. This study further confirmed that the combination of the CWT and RF models has significantly better predictive performance than that of the SVM and GBDT models (Fig 8). Meanwhile, our research also found that the combination of FD and WT for SSC estimation helps improve the prediction accuracy and generalization ability of the model. Especially in the (1/*R*)′-CWT-2^7^ processing, the *R*^2^ values of the test set are all above 0.62, and the RPD values are all greater than 1.48 (Fig 8). Jiang et al. [83] found in their research on predicting SOC content based on RF, SVM, and PLSR algorithms that the SVM model constructed by combining FD and CWT had the highest prediction accuracy. This conclusion is consistent with the result of this study, namely that the SVM model constructed by combining FD and CWT is superior to the RF and GBDT models. Zhang et al. [84] found that the RF model constructed based on the 6th decomposition scale of DWT can effectively predict SOM. This study also found that the RF model constructed based on the H7-scale wavelet coefficients of DWT has better prediction accuracy for SSC than the GBDT and SVM models (Fig 8). Furthermore, the DF model introduced in this study integrates the advantages of the RF model and deep learning, further improving the prediction accuracy and stability of the model. Moreover, the average values of *R*^2^, RMSE, and RPD in the test set of the DF model constructed based on different transformed spectra are 0.64, 17.726 g·kg^-1^, and 1.67, respectively (Fig 8b). Fei et al. [85] also confirmed in their study on predicting wheat yields in different growing seasons using hyperspectral reflectance data that the DF algorithm outperforms the RF algorithm in most spectral transformations. Liu et al. [86] compared the performance of DF, RF, SVM, ANN, and RNN algorithms in tungsten ore prediction. The results showed that the DF model had the highest comprehensive accuracy and the best prediction effect, further verifying the superiority of the DF model. In conclusion, the DF model constructed by integrating FD and WT shows significant advantages in both prediction accuracy and robustness.

This study, based on field sampling data, constructed a methodological framework for monitoring soil salinity in arid oasis areas. This framework consists of three main components: (1) By collecting soil samples, the salt content of the soil and the corresponding spectral data were obtained; (2) Perform various transformations on the spectral data to enhance the salinity-sensitive features and further select the most sensitive feature bands for salinity; (3) Build multiple machine learning models and select the optimal one for soil salt content prediction by comparing the prediction accuracy of the models. By integrating multiple spectral transformations and multiple models, this framework can effectively improve the prediction accuracy of soil salinity content. Existing studies have also shown that combining multiple spectral preprocessing methods with multiple models can enhance the prediction accuracy [2,56]. Therefore, the results of this study provide a systematic method path for regional soil salinity content prediction, and offer a scientific basis for assessing salinization risks and land degradation management in arid areas. However, this study mainly relied on soil reflectance data and did not systematically consider the effects of soil moisture content, texture, surface roughness, and background conditions, which may limit the prediction accuracy to some extent. Future research could integrate multi-source data (e.g., optical images, microwave remote sensing data, surface environmental factors) with multiple algorithms to further improve the accuracy and reliability of predictions [64].

## Conclusion

The following important conclusions are drawn in this study: (a) Correlation analysis indicates that WT and its combinations with mathematical transformations can significantly enhance the correlation between spectral data and SSC. Among these, *R*-DWT exhibits the highest correlation (|*r*|_max_ = 0.621), followed by (1/*R*)′-DWT (|*r*|_max_ = 0.603), (1/*R*)′-CWT (|*r*|_max_ = 0.598), and *R*-CWT (|*r*|_max_ = 0.592). In contrast, FDs exhibited relatively lower correlation, though still markedly higher than *R* (|*r*|_max_ = 0.227). (b) The characteristic bands of SSC are primarily concentrated in the clay mineral absorption band near 1633 nm, the hydrated ion absorption bands at 1809–1810 nm and 1951–1955 nm, and the crystalline water and hydroxyl absorption bands at 1969–1971 nm and 1987–1989 nm. Additionally, there are multiple weak absorption bands related to soluble salts within 2001–2012 nm, 2021–2026 nm, 2029–2034 nm, and 2038–2041 nm. (c) The model prediction results indicate that different spectral transformations have a significant impact on the prediction accuracy of SSC. Among them, the model constructed by the (1/*R*)′ has relatively poor overall performance, with the average *R*^2^ values of the training set and test set being 0.60 and 0.54 respectively. After wavelet transformations, the (1/*R*)′-CWT-2^7^ and *R*-DWT-H7 transformations improved the prediction performance, with the average *R*^2^ of the training set increasing by 0.19 and 0.15, and the average *R*^2^ of the test set increasing by 0.10 and 0.08 respectively. Among all the models, the *R*-DWT-H7-DF has the best comprehensive performance, with the *R*^2^ values of the training set and test set reaching 0.87 and 0.67 respectively; followed by the (1/*R*)′-CWT-2^7^-SVM, with the *R*^2^ values of the training set and test set being 0.76 and 0.67 respectively.
